# Macrocephaly and Digital Anomalies Expand the Phenotypic Spectrum of *PGAP2* Variants in Hyperphosphatasia with Impaired Intellectual Development Syndrome 3 (HPMRS3)

**DOI:** 10.1155/2024/5518289

**Published:** 2024-01-05

**Authors:** Seda Susgun, Afif Ben-Mahmoud, Franz Rüschendorf, Bonsu Ku, Syeda Iqra Hussain, Solveig Schulz, Oliver Puk, Saskia Biskup, Jonathan D. J. Labonne, Dilan Wellalage Don, Vijay Gupta, Tae-Ik Choi, Saadullah Khan, Naveed Wasif, Yves Lacassie, Lawrence C. Layman, Sibel Aylin Ugur Iseri, Cheol-Hee Kim, Hyung-Goo Kim

**Affiliations:** ^1^Department of Biology, Chungnam National University, Daejeon 34134, Republic of Korea; ^2^Department of Genetics, Aziz Sancar Institute of Experimental Medicine, Istanbul University, Istanbul, Türkiye; ^3^Neurological Disorders Research Center, Qatar Biomedical Research Institute, Hamad Bin Khalifa University, Doha, Qatar; ^4^Max Delbrück Center for Molecular Medicine in the Helmholtz Association, Berlin, Germany; ^5^Disease Target Structure Research Center, Korea Research Institute of Bioscience and Biotechnology (KRIBB), Daejeon 34141, Republic of Korea; ^6^Department of Biotechnology and Genetic Engineering, Kohat University of Science & Technology (KUST), Kohat, Khyber Pakhtunkhwa, Pakistan; ^7^Zentrum für Humangenetik, Tübingen, Germany; ^8^Center for Genomics and Transcriptomics (CeGaT), Tübingen, Germany; ^9^SalioGen Therapeutics, Lexington, MA, USA; ^10^Institute of Human Genetics, University of Ulm, Ulm, Germany; ^11^Institute of Human Genetics, University Hospital Schleswig-Holstein, Campus Kiel, Kiel, Germany; ^12^Department of Pediatrics, Louisiana State University Health Sciences Center, New Orleans, LA, USA; ^13^Section of Reproductive Endocrinology, Infertility and Genetics, Department of Obstetrics and Gynecology, Augusta University, Augusta, GA, USA; ^14^Department of Neuroscience and Regenerative Medicine, Augusta University, Augusta, GA, USA; ^15^College of Health and Life Sciences, Hamad Bin Khalifa University, Doha, Qatar

## Abstract

Glycosylphosphatidylinositols (GPIs) anchor over 150 proteins as GPI-anchored proteins (GPI-APs) with crucial roles in diverse biological processes. The highly conserved biosynthesis of GPI-APs involves precise steps with at least 21 genes, categorized as *PIG* and *PGAP* genes. Pathogenic variants in these genes are linked to human diseases, highlighting the importance of each biosynthesis step. *PGAP2* stands out among these genes due to its association with an expanded clinical spectrum of neurodevelopmental disorder (NDD) phenotypes with biallelic pathogenic variants. We present four patients from two families, one consanguineous and the other nonconsanguineous, each displaying distinct clinical presentations, including intellectual disability, hyperphosphatasia, hearing impairment, and epilepsy, as well as craniofacial and digital anomalies. Genetic analyses revealed homozygous and novel compound heterozygous missense variants in *PGAP2* in four affected individuals, confirming the molecular diagnosis of hyperphosphatasia with impaired intellectual development syndrome 3 (HPMRS3). Importantly, the three amino acids affected by missense variants exhibit complete conservation in 10 vertebrate species, illuminating their crucial role in the gene's functionality. Protein modeling provided additional evidence for the pathogenicity of the three substitutions, demonstrating their detrimental impact on protein folding and putative protein-protein interactions, ultimately leading to impaired protein function. The four patients in our study displayed common phenotypic features, such as brachydactyly, camptodactyly, and syndactyly, which have not been previously documented in individuals with *PGAP2* variants. Notably, the occurrence of macrocephaly in two affected brothers from a consanguineous Pakistani family represents a novel finding. These previously unreported digital anomalies, along with macrocephaly and the identification of novel compound heterozygous variants, contribute to the expansion of the phenotypic and genotypic spectrum of HPMRS3 associated with *PGAP2* variants.

## 1. Introduction

Glycosylphosphatidylinositols (GPIs) are glycolipids that serve as anchors for over 150 proteins, forming GPI-anchored proteins (GPI-APs). GPI-APs are present on the cell surface and play essential roles in various biological processes, including membrane protein trafficking, cell-surface adhesion, and signal transduction [1–4]. The biosynthesis of GPI-APs is highly conserved across eukaryotes and involves multiple precise steps to generate functional GPI-APs [3]. This process involves at least 21 genes, which can be categorized into two main groups: Phosphatidyl Inositol Glycan (*PIG*) genes and Post-GPI Attachment to Proteins (*PGAP*) genes [4, 5]. Each step of GPI-AP biosynthesis is crucial, as pathogenic variants in nearly all *PIG* and *PGAP* genes have been implicated in human diseases [2].

**Figure 1:**
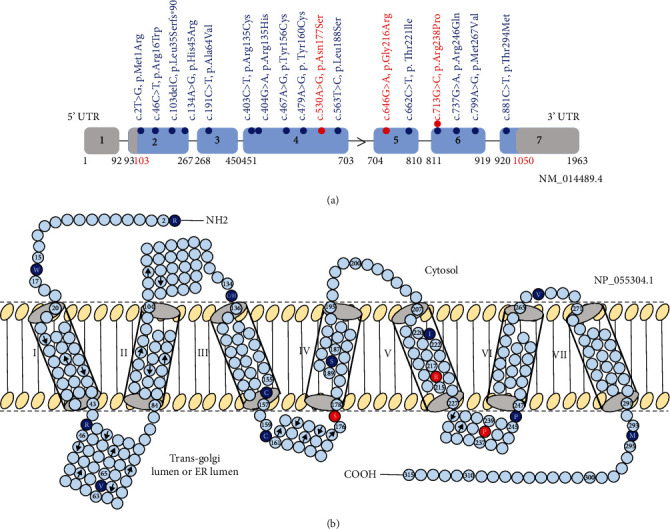
Visual representation of previously published variants (depicted in blue letters) and our identified variants (depicted in red letters) in *PGAP2*, illustrating their locations at both the cDNA and protein levels. (a) An overview of the distribution of *PGAP2* mutations at the cDNA level, utilizing the exon/intron structure of *PGAP2* mRNA (NM_014489.4). Reported mutations, excluding one found in our patients, are depicted in blue and include 12 missense mutations and one frameshift mutation. The three variants identified in our study, including one reported variant (with blue and red balls) and two novel variants, are highlighted in red. Specifically, the variant c.713G>C has been previously documented in two published papers. This brings the total reported mutations to 14 (13 missense and one frameshift mutations). (b) A topology diagram of the amino acid sequence of the human PGAP2 protein NP_055304.1 that was generated using the InterPro database server (https://www.ebi.ac.uk/interpro/) [12]. The predicted seven transmembrane domains (TMDs) are indicated as cylinders, numbered from I to VII. TMD I: 20-43; TMD II: 84-104; TMD III: 136-157; TMD IV: 178-195; TMD V: 207-227; TMD VI: 247-265; TMD VII: 271-291. Also shown are the predicted extracellular loops above and the intracellular loops below the TMDs. All reported missense mutations are depicted in blue circles with mutated amino acid abbreviations. Three missense variants we identified are depicted in red circles with mutated amino acid abbreviations. Residue numbers are given at the boundary of TMDs and at other locations near mutated residues.

Among these genes, *PGAP2* (Post-GPI Attachment to Proteins 2, MIM 615187) is involved in the remodeling of lipid components of GPI anchors in the Golgi [6, 7] and endoplasmic reticulum (ER) (Figure 1(b)) [8]. Biallelic pathogenic variants in *PGAP2* have been associated with hyperphosphatasia with impaired intellectual development syndrome 3 (HPMRS3, MIM 614207) [3, 7, 9–11].

We enrolled two independent families with a total of four affected members who received a provisional clinical diagnosis of HPMRS based on hyperphosphatasia and intellectual disability (ID) among other phenotypes. Through our genetic analysis and protein modeling, we successfully identified three distinct missense variants in *PGAP2*, thereby establishing a molecular diagnosis of HPMRS3 for these individuals (Table 1).

One of these variants, previously reported in two different consanguineous Pakistani families, was found in our consanguineous Pakistani family 1. Given the shared ethnic background, we wanted to determine whether this same variant in *PGAP2* resulted in a concordant or discordant phenotype. In family 2, we observed novel compound heterozygous missense variants, representing a previously unreported genotype. These variants have the potential to give rise to a novel phenotype, especially when a nonconsanguineous German family carrying *PGAP2* variants was not documented so far.

As the phenotypic spectrum of HPMRS3 is heterogenous and continues to expand with new cases described in the literature, we compared the detailed phenotypes of our four patients amongst themselves. Furthermore, we compared their phenotypes with those reported in patients harboring both the same and different *PGAP2* variants (Supplementary Table 1). Our aim was to investigate whether a genotype-phenotype relationship could be established for the *PGAP2* gene.

## 2. Subjects and Methods

### 2.1. Subjects

**Figure 2:**
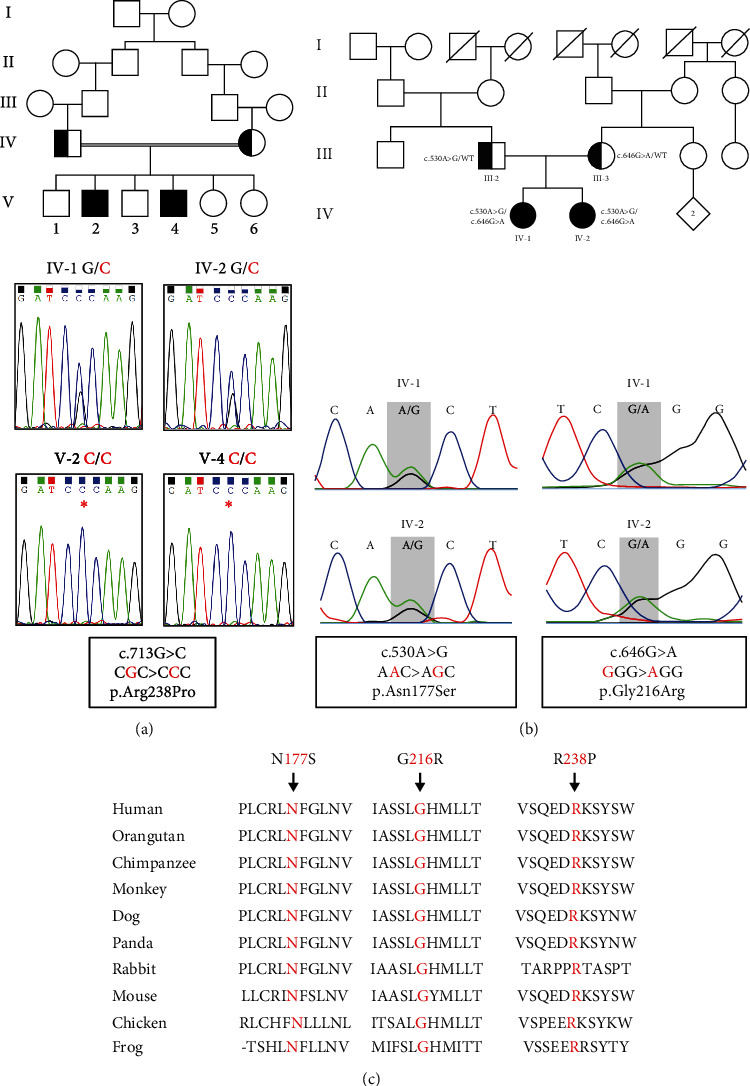
The pedigrees of two families with biallelic variants in *PGAP2*, resulting in the alteration of three conserved residues across ten vertebrate orthologs. (a) In the consanguineous family 1 originating from Pakistan, two affected brothers were identified, both of whom carried a homozygous missense variant c.713G>C, resulting in the p.Arg238Pro substitution. This variant was confirmed in the chromatogram and was found to be segregated within the family. (b) Family 2, with nonconsanguineous parents, had two affected daughters who carried compound heterozygous variants: c.530A>G, p.Asn177Ser, c.646G>A, and p.Gly216Arg. These variants were detected in the chromatogram and segregated within the family. (c) Conservation of three crucial amino acids in PGAP2 across ten vertebrate species. The protein sequence alignment revealed that three amino acids (N177, G216, and R238) in PGAP2, which were impacted by missense variants in our patients and marked in red, were fully conserved among all ten vertebrate species.

Two families, one of Pakistani origin and the other of German origin, were enrolled in this study (Figures 2(a) and 2(b)). Comprehensive physical and neurological examinations were conducted, and detailed family histories were obtained through consultations. Written informed consents were obtained from all participants, in accordance with the Helsinki-II Declaration. This study protocol was approved by the Ethics Committee of Kohat University of Science and Technology (KUST/Ethical Committee/16-25) and the Institutional Review Board of Augusta University, Georgia, USA.

### 2.2. Family 1

#### 2.2.1. Exome Sequencing and *In Silico* Variant Analysis

Genomic DNA (gDNA) was extracted from whole blood samples of the two affected individuals (V-2 and V-4; Figure 2(a)). The gDNA of the probands was subjected to exome sequencing. For the exome library preparation, the TruSeq Exome Enrichment kit (Illumina, San Diego, CA, USA) was utilized following the manufacturer's instructions. Paired-end sequencing (2 × 76 bp) was performed on the NextSeq500 sequencer (Illumina, San Diego, CA, USA) using a v2 high-output reagent kit (Illumina). The obtained data were aligned to the human reference genome GRCh37 (hg19). *In silico* variant analysis and variant prioritization were conducted using previously established methods and protocols [13]. We utilized a comprehensive set of techniques to evaluate the pathogenicity of variants identified in the VCF files. To generate the initial list of candidate variants, we applied a series of filtering steps. These steps involved including variants with a frequency below 0.1% in ExAC/gnomAD v2.11/1000g2015, focusing on exonic, splicing, nonsynonymous, and stop-gain variants with sufficient coverage, discarding variants predicted to be benign or tolerated in PolyPhen and SIFT, and retaining variants with a CADD score ≥ 20. Moreover, we conducted further filtering using annotation data from public databases such as the Mouse Genome Database (MGD), OMIM, PubMed, and ClinVar. Our selection of final variants was guided by ACMG guidelines, absence in control datasets (gnomAD), relevance of interacting proteins in neurodevelopmental disorders and intellectual disability based on literature, presence of sporadic variants in the Human Gene Mutation Database (HGMD), expression patterns in human tissues, and phenotypes observed in available knock-out/deletion model organisms. To assess the impact of missense variants on the structure and functionality of our candidate genes, we employed protein modeling. Through this rigorous procedure, we successfully identified one homozygous variant in PGAP2, recessively inherited from both heterozygous parents. The candidate variants and their familial segregation were subsequently confirmed using Sanger sequencing. To ensure accurate variant annotation, all identified variants underwent reannotation using the Ensembl Variant Effector Predictor (VEP) tool [14].

#### 2.2.2. Genotyping and Quality Control

The Illumina Infinium HumanCore-24 v1.0 Bead Chip array (Illumina, Inc., San Diego, CA, USA) was used to genotype eight individuals in this family, including the parents and 6 offspring. Over 300k markers on the array were initially considered, but indels, MT- and Y-chromosomal SNPs, and variations without physical positions were filtered out. This resulted in a maximum of 259,460 biallelic SNPs before further quality control (QC) and linkage analysis. ALOHOMORA software [15] was utilized for data conversion to linkage format files and QC. The sex of individuals was determined by comparing the count of heterozygous genotypes on the X chromosome to the known pedigree data. The program Graphical Relationship Representation (GRR) [16] was employed to verify the relationships between family members. To detect Mendelian errors (ME), PedCheck was used [17], and any SNPs with ME were removed from the dataset. Unlikely genotypes, such as double recombinants, were identified with Merlin [18] and subsequently removed from the individuals.

#### 2.2.3. Autozygosity Mapping

Autozygosity mapping, a type of linkage analysis, was performed using Merlin software with an autosomal recessive mode of inheritance and complete penetrance. To calculate allele frequencies of genetic markers, data from 491 Pakistani individuals were utilized. At the disease locus, a minor allele frequency of 0.001 was assigned. Instead of a genetic map for marker distances, we used the converted physical distances, where 1 Mb was equivalent to 1 cM. Two separate analyses were conducted using Merlin. The first analysis involved a full marker set comprising approximately 258,300 SNPs after QC. This step was aimed at determining the optimal positions for recombination events. The second analysis employed a reduced marker set of around 119,400 SNPs, with a minimum distance of 10,000 bases between markers. By reducing the linkage disequilibrium (LD) between markers, this analysis identified inflated linkage peaks caused by markers in LD. Linkage regions where the LOD score decreased by more than 0.3 in the LD-reduced analysis were removed. In summary, regions were selected based on reaching the maximum LOD score in this family and exhibiting stable LOD scores in the less dense LD-reduced marker set. Given the recessive inheritance model and the presence of a consanguinity loop (a second-order cousin marriage) within the pedigree structure, this linkage analysis is referred to as autozygosity mapping.

### 2.3. Family 2

#### 2.3.1. Epilepsy Gene Panel Analysis

Starting from EDTA blood, genomic DNA was isolated according to the manufacturers' instructions using a QIAamp DNA Blood Maxi Kit on a QiaSymphony instrument (Qiagen, Hilden, Germany). DNA quantity and quality were determined using Qubit® Fluorometer and NanoDrop ND-8000 (Thermo Fisher Scientific, Dreieich, Germany). Sequencing libraries were prepared for each sample from 50 ng DNA using the Twist enrichment workflow (Twist Bioscience, San Francisco, CA) and a custom-design enrichment probe set (CeGaT ExomeXtra V3). The enrichment was restricted to targets (gene regions) that were associated with epilepsy and brain development disorders at the time. The panel contained 635 genes. Library preparation and capture were performed according to the manufacturer's instructions, and paired-end sequencing was performed on a NovaSeq6000 instrument (Illumina, San Diego, CA) with 2 × 100 base pair (bp) read length. Sequence data were processed with Illumina bcl2fastq2. Adapter sequences were removed with Skewer, and the sequences obtained were aligned to the human reference genome (hg19) with the Burrows-Wheeler Aligner (BWA mem). Sequences that could not be clearly assigned to a genomic position were removed, as were sequence duplicates that were probably due to amplification (internal software). Mean coverage on target for this sample was 810.605. Sequence variants (single nucleotide exchanges and short insertions/deletions) were determined from the remaining high-quality sequences (CeGaT StrataCall). Resulting variants were annotated with population frequencies from gnomAD (v2.1/3.1) and an internal database (CeGaT), factoring in external databases (e.g., HGMD and ClinVar), and with transcript information from Ensembl, RefSeq, Gencode, and CCDS. All variants were manually assessed before inclusion in the final report, classified, and reported based on ACMG/ACGS-2020v4.01 guidelines.

#### 2.3.2. Protein Modeling

To investigate the impact of the N177S, G216R, and R238P substitutions on the PGAP2 protein, atomic level analysis was conducted. As the experimental structure of PGAP2 has not been determined, a three-dimensional structural model was generated using AlphaFold prediction (https://alphafold.ebi.ac.uk/entry/Q9UHJ9).

## 3. Results

### 3.1. Clinical Presentation and Molecular Diagnosis

#### 3.1.1. Family 1

In a Pakistani family, six children were born from two healthy Pakistani parents, who are first cousins (Figure 2(a)). Among them, the second and fourth males are affected by severe syndromic ID. These two Pakistani brothers are currently 25 and 20 years old, respectively. The remaining elder and younger siblings, composed of two brothers and two younger sisters, are unaffected. Exome sequencing was performed on the two affected brothers, which identified a homozygous missense variant c.713G>C (NM_014489.4), p.Arg238Pro (NP_055304.1), in *PGAP2* in both patients (Figures 1 and 2(a) and Table 1). This variant was found within the previously identified 8.98 Mb homozygous genomic stretch on chromosome 11, as determined by autozygosity mapping. The presence of this variant within the autozygous region further supported its pathological role in the autosomal recessive genetic disorder based on the consanguineous pedigree. It was reported in gnomAD (9/251,414 alleles) as pathogenic with a CADD score of 23.6, predicting to be deleterious (Table 1). Sanger sequencing confirmed the presence of homozygous variants in two affected brothers (V-2 and V-4). Additionally, it revealed that both parents (IV-1 and IV-2) and three unaffected available siblings (V-3, V-5, and V-6) are all carriers of this variant in a heterozygous state. The observed segregation pattern within the family aligns with an autosomal recessive inheritance pattern (Figure 2(a)), thus confirming the diagnosis of HPMRS3.

Two brothers presented with severe ID. Additionally, they are unable to pronounce words or form sentences, only producing specific sounds when their mother is in front of them. Communication of their daily needs, such as food, changing clothes, or using the toilet, is not possible for them without their mother or any caregiver. Aside from their mother, they show no response to any other family members and produce unclear sounds when they experience pain or injury. These brothers have a hearing deficiency and do not respond to sounds ranging from low to loud. Moreover, there is no reaction when their mother calls them by their names. Further investigations included tympanometry, which revealed bilateral hearing impairment ranging from severe to profound in both patients. They are unable to walk in a straight line. During the physical examination, no bone deformities were observed in the patients.

**Figure 3:**
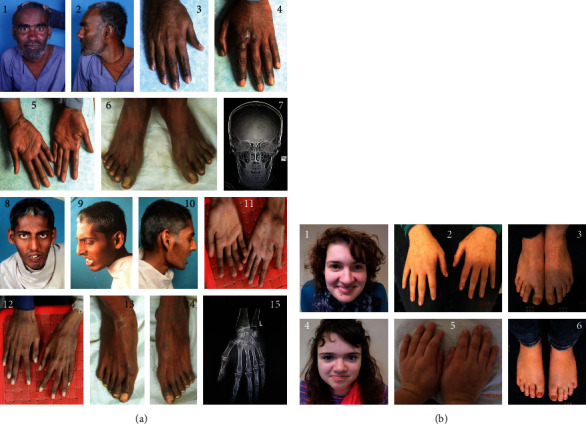
Photographs depicting the craniofacial and limb features of individuals with biallelic variants in *PGAP2*. (a) Photographs of family 1, featuring elder brother V-2 in photos 1 to 7 and his younger brother V-4 in photos 8 to 15. Subject V-2 displays macrocephaly and some facial asymmetry (1). He also presents fluent eyebrows with synophrys and a tendency towards telecanthus/hypertelorism (1) as well as an appearance of bifid tragus (2). Brachydactyly of the 5th fingers is observed (3, 4, and 5), along with tapering fingers (3 and 4). A bifurcation of the transverse proximal palmar flexion crease is seen in the palm (5). Camptodactyly in the 3rd left toe and 5th right toe is evident (6). X-ray of the head confirms macrocephaly (7). Subject V-4 presents macrocephaly with a triangular-shaped head, mild synophrys, telecanthus with hypertelorism, and distinctive eyes (small irises compared to enlarged eyeballs) (8, 9, and 10). Hypoplasia of the thenar areas is observed in the palms with no apparent vertical palmar flexion creases (11). Bilaterally, there is minor syndactyly between fingers 2 and 3 and tapering fingers (12), and camptodactyly of the 5th right toe is present (13). X-ray of the left hand exhibits mild soft tissue syndactyly between the 2nd and 3rd fingers, as well as between the 3rd and 4th fingers (15). Similar to his elder brother V-2 (X-ray not shown), the distal phalanges of the 2^nd^, 3^rd^, and 4^th^ fingers also appear slightly shorter (15). (b) Photographs depicting family 2 show the elder sister, IV-1, in photos 1 to 3, and her younger sister, IV-2, in photos 4 to 6. The elder sister IV-1 exhibits a prominent forehead with a pointed chin, along with telecanthus/hypertelorism (1). Dorsal view of the hands reveals bilateral shortening of the 5th fingers along with tapering fingers (2). Dorsal view of the feet shows a bilateral tendency to camptodactyly in toes 4 and 5 (3). The younger sister IV-2 exhibits similar facial features, including a prominent forehead, pointed chin, telecanthus/hypertelorism, and a broad nose with a wide nasal tip (4). Both hands show tapering fingers and noticeable shortening of the 5th finger (5). Dorsal view of the feet reveals a gap between the halluces and 2nd toes, along with a tendency towards clino/camptodactyly of toes 4 and 5, and possible syndactyly of toes 2 and 3 on the right foot (6).

The elder affected brother (V-2) is currently 25 years old. He has a height of 167.64 cm, which falls between the 5th and 10th percentile on the NCHS growth chart. His head circumference (OFC) measures 58 cm, which is above the 98th percentile for adults according to the Nellhaus growth chart, indicating macrocephaly (Figure 3(a): 1, 2, and 7). He has some facial asymmetry, prominent eyebrows with synophrys and a tendency towards telecanthus/hypertelorism, with eyelid spacing at 10 and intercanthal distance at 12 (Figure 3(a): 1). There appears to be some indication of a bifid tragus (Figure 3(a): 2). Camptodactyly is present in the 3rd left toe and 5th right toe (Figure 3(a): 6). He has brachydactyly (shortened fifth finger) and tapering fingers on both hands (Figure 3(a): 3, 4, and 5). Similar to his younger brother V-4 (Figure 3(a): 15), the X-ray of V-2's left hand indicates slight shortening of the distal phalanges in the 2nd to 4th fingers (X-ray not shown). Additionally, a bifurcation of the transverse proximal palmar flexion crease was observed on the right palm (Figure 3(a): 5). Serum alkaline phosphatase levels were significantly elevated reaching 743 U/L, compared to the reference range of 50-116 U/L.

The younger affected brother (V-4) is currently 20 years old. He has a height of 170.18 cm, which falls between the 10th and 25th percentile. His head circumference measures 57 cm, just below the 98th percentile for height, indicating a slight tendency towards macrocephaly (Figure 3(a): 8, 9, and 10). He has a triangular face along with synophrys (Figure 3(a): 8). The presence of telecanthus with hypertelorism is observed with eyelid spacing at 9 and an intercanthal distance of 11 (Figure 3(a): 8). Distinct eyes with small irises in relation to enlarged eyeballs are noticeable (Figure 3(a): 8). There is a hypoplasia of the thenar areas and the absence of vertical palmar flexion creases (Figure 3(a): 11). Furthermore, the hands exhibit bilateral, minor syndactyly between fingers 2 and 3, along with tapering fingers (Figure 3(a): 12). Additionally, the 5^th^ right toe shows camptodactyly (Figure 3(a): 13). The left-hand X-ray confirmed mild soft tissue syndactyly between the 2nd and 3rd fingers, along with additional syndactyly between the 3rd and 4th fingers (Figure 3(a): 15). Additionally, the X-ray revealed slight shortening of the distal phalanges in the 2^nd^, 3^rd^, and 4^th^ fingers of the left hand (Figure 3(a): 15). An alkaline phosphatase test was not available for him.

*(1) Autozygosity Mapping Results*. Autozygosity mapping revealed two genomic regions of long homozygous stretches, where the LOD score reached the maximum LOD 2.64 and is stable in the analysis with the LD-reduced marker set.

The genomic coordinates of the homozygous candidate gene region in GRCh37/hg19 are as follows: Chr 11: 2182224-11161248, length: 8,979,924 bp, and Chr 17: 67057288-76472768, length: 9,415,480 bp.

#### 3.1.2. Family 2

Below, we present two female siblings of German origin, born to nonconsanguineous parents (Figure 3(b)). They exhibit refractory epilepsy, ID, and hyperphosphatasia. During the initial consultation, the older sister (IV-1) was 23 years old (Figure 2(b)). She was born after a spontaneous vaginal delivery at 40 weeks' gestation, weighing 3.2 kg. Her birth length was 51 cm, and her head circumference was 34.5 cm. She was able to sit independently at 6 months, began walking at 15 months, and uttered her first words at 24 months. However, she did not start forming complete sentences until the age of 3.5 years. The first seizure occurred when she was 2 years old and initially presented as epilepsy, manifesting as complex-partial seizures and absence-like states. By the age of 11 years, she has developed epilepsy with generalized seizures accompanied by focal signs, which could not be clearly classified at that time. Severe ID was also observed. The electroencephalogram (EEG) showed recurring generalized epilepsy-like potentials, with a frontal maximum of 3-4 Hz per spike-wave complex lasting up to 4 seconds. Additionally, her alkaline phosphatase levels were repeatedly elevated, reaching up to 1237 U/L. Despite attempts with various medications, including valproate, sultiam, clobazam, ethosuximide, and fortecortin, the epilepsy remained resistant to treatment. She displays craniofacial anomalies such as a prominent forehead, a pointed chin, and mild telecanthus/hypertelorism (Figure 3(b): 1). Moreover, her hands exhibit tapering fingers and bilateral shortening of the 5th fingers (Figure 3(b): 2), while her feet show a tendency towards bilateral camptodactyly in toes 4 and 5 (Figure 3(b): 3). Her body mass and head circumference are within the normal range.

During the first consultation, the younger sister (IV-2) was 19 years old (Figure 2(b)). She was born at full-term through a spontaneous vaginal delivery, weighing 3.2 kg, with a birth length of 50 cm and a head circumference of 34.5 cm. Her early childhood development appeared normal without any notable concerns. At the age of 3 years, she experienced the onset of epilepsy. By the age of 6, significant attention and concentration deficits were observed, along with ID indicated by an IQ score of 48. Her epilepsy manifested as generalized seizures, absence seizures, and tonic-clonic seizures, occurring at a frequency of 1 to 10 per month. Unfortunately, epilepsy proved resistant to treatment with valproate, sultiam, and ethosuximide. Elevated alkaline phosphatase levels, reaching up to 1850 U/L, were detected. Cranial MRI did not reveal any pathological findings. However, the awake EEG displayed severe abnormalities, including groups of generalized frontal accentuated spike-wave complexes around 3 Hz, which intensified during hyperventilation with right frontal accentuation. Additionally, discontinuous slowing was observed in the centroparietal region on the right side, with PU over P4, indicative of abnormalities in all leads. The EEG also displayed an alpha-basal rhythm. These findings strongly support a diagnosis of generalized epilepsy. She exhibits similar facial features, including a prominent forehead, pointed chin, and telecanthus/hypertelorism, along with a broad nose and wide nasal tip (Figure 3(b): 4). Both of her hands display tapering fingers and apparent shortening of the 5th finger (Figure 3(b): 5). Additionally, her feet show a gap between the halluces and 2nd toes, a tendency towards clino/camptodactyly in toes 4-5, and a possibility of syndactyly between toes 2 and 3 on the right foot (Figure 3(b): 6). Gene panel analysis was conducted using a panel consisting of 635 known epilepsy genes. This analysis revealed the presence of novel compound heterozygous variants in *PGAP2*, c.530A>G (NM_014489.4), p.Asn177Ser, and c.646G>A, p.Gly216Arg, in the elder sister IV-1. The segregation analysis by Sanger sequencing revealed that the affected younger sister IV-2 shares the same compound heterozygous variants. The father carried the heterozygous variant c.530A>G, while the mother carried the other heterozygous variant c.646G>A. This confirms the segregation of compound heterozygous variants in the family, with each parent being a carrier of a different variant, thereby validating the pathological role of biallelic variants in *PGAP2* and confirming the diagnosis of HPMRS3 (Figures 1 and 2(b)).

### 3.2. Genetic Analyses

ES and epilepsy gene panel analyses identified three missense variants in *PGAP2* in four affected individuals in both families. The variants were reannotated using Ensembl VEP, specifically as ENST00000278243.9/NM_014489.4, based on the MANE Select transcript. To confirm the inheritance pattern, Sanger sequencing was conducted, which revealed that the variants segregate within the family, consistent with an autosomal recessive inheritance model (Figures 2(a) and 2(b)). Detailed information on the presented variants can be found in Table 1. Furthermore, Figure 1 visually presents all previously published variants, along with our identified variants, in *PGAP2* at both the cDNA and protein levels (Supplementary Table 1).

### 3.3. Molecular Modeling of N177S, G216R, and R238P Substitutions

**Figure 4:**
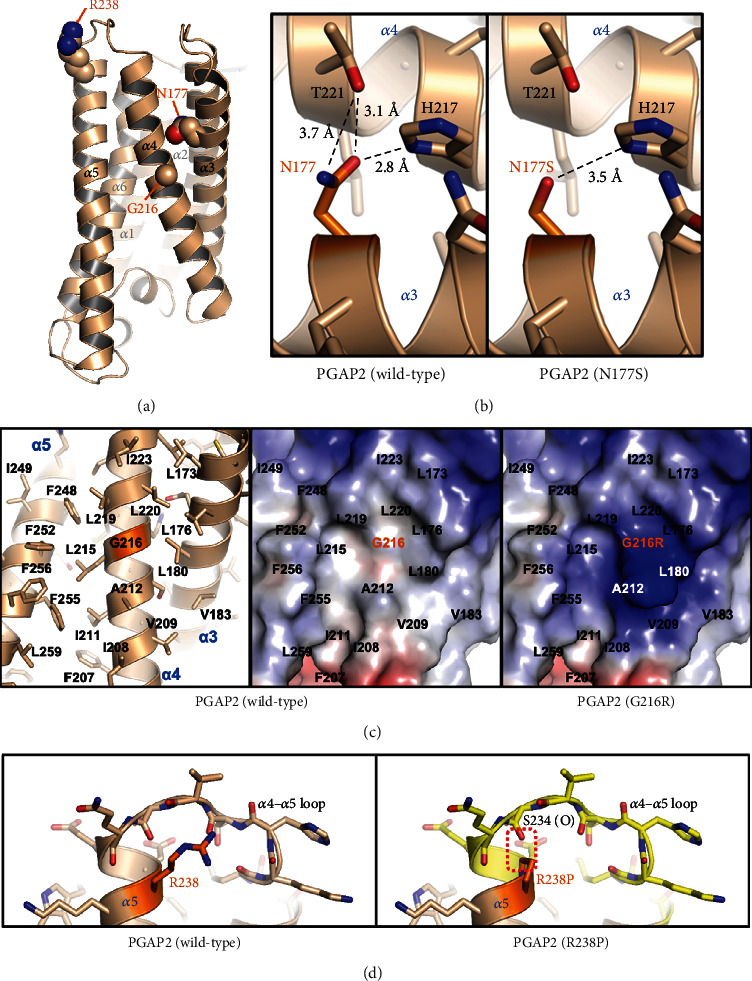
Structural effects of three *PGAP2* missense variants through protein modeling. (a) Asn177, Gly216, and Arg238 are indicated as spheres on the secondary structure-labeled PGAP2 structure modeled using AlphaFold. (b) Interior misfolding due to N177S substitution. Wild type (left) and the N177S (right) mutant form are shown together. Dashed lines indicate hydrogen bonding mediated between Asn/Ser177, Thr221, and His217. The side chain of Asn177 plays a crucial role in maintaining proper protein folding by participating in an intramolecular hydrogen bonding network with His217 and Thr221. The substitution of Asn177 with Ser is expected to disrupt this network, leading to the misfolding of PGAP2. (c) Alteration of protein shape and surface charge due to the G216R variant. PGAP2 wild type is shown as sticks and cartoon (left) or electrostatic surface representation (middle). PGAP2 G216R is presented as electrostatic surface representation (right). Hydrophobic residues consisting of the putative protein-interacting large flat surface are labeled. Gly216 is positioned at the center of a hydrophobic surface formed by *α*3, *α*4, and *α*5 of PGAP2 (left and middle), which is thought to serve as a binding interface for an unknown interacting partner. The substitution of Gly216 with arginine significantly alters the surface's shape and charge distribution (right), likely disrupting potential protein-protein interactions involving PGAP2. (d) Introduction of proline substitution (right) into the wild-type PGAP2 modeling (left). Steric hindrance between C_*δ*_ of the substituted proline and the main chain carbonyl of Ser234 is marked with a dashed rectangle. The region that is assumed to undergo a conformational transition or movement because of the R238P variant is presented in yellow (right). Arg238 is positioned at the N-terminal of the *α*5 helix (see (a)). Replacement of this arginine with proline in the PGAP2 model causes significant steric hindrance between the C*δ* of Pro238 and the main chain carbonyl of Ser234, resulting in partial destabilization of the PGAP2 structure. This structural alteration will impact its functionality.

In the PGAP2 model, the side chain of Asn177 is positioned towards the interior of the protein (Figure 4(a)) and contributes to the proper protein folding by forming an intramolecular hydrogen bonding network with His217 and Thr221 (Figure 4(b)). The substitution of Asn177 with Ser177 is presumed to weaken this network, resulting in instability of PGAP2 protein folding (Figure 4(b)).

Gly216 is centrally located on a large flat surface comprising hydrophobic residues from *α*3, *α*4, and *α*5 of PGAP2 (Figure 4(c), left and middle). This surface is believed to function as a binding interface for an as-yet-undetermined interacting partner. Substituting this residue with arginine has been shown to critically alter the shape and charge distribution of the surface (Figure 4(c), right), likely resulting in the abrogation of putative protein–protein interactions involving PGAP2. Arg238 is located at the N-terminal end of the *α*5 helix (Figure 4(a)). Substituting this arginine with proline in the PGAP2 model results in severe steric hindrance between C_*δ*_ of the substituted proline and the main chain carbonyl of Ser234, with a distance of 1.8 Å (Figure 4(d)). To resolve this clash, the *α*4–*α*5 loop region needs to be repositioned, which may involve unwinding the initial portion of the *α*5 helix that includes Gln235, Glu236, Asp237, and Pro238. Furthermore, these residues participate in intramolecular associations with other PGAP2 residues, supporting proper protein folding. Consequently, it is estimated that the substitution of Arg238 with Pro238 leads to at least partial destabilization of the PGAP2 structure, which certainly affects its functionality. In summary, the protein modeling suggests that the identification of three substitutions, Asn177Ser, Gly216Arg, and Arg238Pro, has deleterious consequences on the structure and stability of PGAP2, thereby negatively impacting its functionality.

## 4. Discussion

Hyperphosphatasia with mental retardation syndrome (HPMRS), commonly referred to as Mabry syndrome, is a rare genetic disorder characterized by elevated levels of alkaline phosphatase in the blood and ID [19]. This condition exhibits genetic heterogeneity, manifesting in five distinct subtypes, each associated with a unique causative gene: HPMRS1, HPMRS2, HPMRS3, HPMRS4, and HPMRS6 are associated, respectively, with mutations in *PIGV*, *PIGO*, *PGAP2*, *PGAP3*, and *PIGY*. Specifically, HPMRS3 represents one of the subtypes within the broader classification of HPMRS, and it is primarily characterized by biallelic mutations in *PGAP2*. However, there have been reports of two heterozygous carriers in siblings with a mild phenotype, including learning disabilities without ID and slightly elevated alkaline phosphatase levels [3].

The GPI anchor synthesis pathway is a cellular process responsible for attaching GPI (glycosylphosphatidylinositol) anchors to proteins. GPI anchors are lipid structures that serve as anchors to attach specific proteins to the outer cell membrane. GPI-AP (GPI-anchored protein) refers to proteins that have a GPI anchor attached to them. These proteins are attached to the outer leaflet of the cell membrane via the GPI anchor. The biosynthesis of GPI precursors involves a meticulously orchestrated series of reactions, leading to the anchoring of the related protein from a diverse set of over 150 different proteins. This stepwise process initiates in the endoplasmic reticulum (ER), and immature GPI-APs are then transported to the Golgi apparatus, where they undergo remodeling and maturation processes. Finally, mature GPI-APs are presented on the plasma membrane [2]. GPI-APs play crucial roles in various biological functions, including cell adhesion, neurogenesis, immune response, and signaling [19].

The regulation of this multistep process is primarily governed by *PIG* and *PGAP* genes, which are conserved across eukaryotes. It is noteworthy that each step of the GPI-AP biosynthesis seems to be indispensable, as biallelic variants in *PIG/PGAP* genes have been implicated in NDDs including DD, ID, epilepsy, congenital malformations, and dysmorphism [2, 4]. A comprehensive study conducted in 2017 reported that *PIG/PGAP* genes (*PGAP3*, *PIGN*, *PIGT*, *PIGO*, and *PIGL*) were found to be responsible for approximately 0.15% of DD cases among 4293 trio exome data [20].

PGAP2, a crucial component in the biosynthesis of GPI-anchored proteins (GPI-APs), facilitates the reacylation process during lipid remodeling in the trans-Golgi apparatus. The longest isoform of PGAP2, isoform 1 (NP_055304.1) consisting of 315 amino acids, is predicted to contain seven transmembrane domains according to the InterPro database (Figure 1(b)) [12]. Comparably, the shorter isoform 8, NP_001243169.1 (254 aa), has five transmembrane domains as predicted by TMHMM [9]. The homozygous variant (c.713G>C, p.Arg238Pro) in isoform 1 of family 1, previously reported as c.530G>C, p.Arg177Pro in isoform 8, is predicted to reside in the same trans-Golgi lumen or ER lumen (Figure 1(b)) [9]. This prediction aligns with another reported mutation (c.296A>G, p.Tyr99Cys) in isoform 8 [9], which is also predicted to be in the trans-Golgi lumen or ER lumen in our isoform 1 (c.479A>G, p.Tyr160Cys) (Figure 1(b)). Of the two compound heterozygous variants in family 2, the alteration p.Asn177Ser (c.530A>G) was predicted to reside in the trans-Golgi lumen or ER lumen, while p.Gly216Arg is predicted to reside in the 5th transmembrane domain (Figure 1(b)). In general, mutations in *PGAP2* are distributed across various regions, including the cell cytosolic and transmembrane domains, as well as the trans-Golgi lumen or ER lumen (Figure 1(b)).

Interestingly, our three variants have been classified as polymorphisms and registered at dbSNP in NCBI, displaying an extremely low or null allele frequency with no reported homozygotes (Table 1). Therefore, caution should be exercised when interpreting genetic variants listed in dbSNP, as they could potentially be disease-causing variants in biallelic patterns in autosomal recessive disorders, as discussed [3]. This caution also extends to X-linked recessive disorders, as the variants listed in dbSNP could originate from asymptomatic carrier females.

While there is an indication that PGAP2 may function as an acyltransferase, further evidence is required to confirm this hypothesis [2]. Mutations in *PGAP2* can lead to functional deficiencies, impairing its enzymatic activity and resulting in incomplete or incorrect modifications of GPI precursors. This disruption interferes with the proper maturation of GPIs, leading to impaired functionality [19]. Consequently, the attachment of GPI anchors to proteins is affected. The modified GPI anchors may fail to effectively anchor GPI-APs to the cell membrane, potentially producing structurally abnormal GPI-APs. The deficiency of functional GPI-APs can cause the alteration or loss of various GPI-APs on the cell surface, impacting their normal functions. The loss of functional GPI-APs in HPMRS3 is thought to play a significant role in the manifestation of neurological and developmental abnormalities in affected individuals.

This notion is further supported by studies involving a mouse model with mutations in *PGAP2*. These studies have demonstrated the essential role of GPI-APs in crucial processes such as neural tube closure, heart development, and the survival of cranial neural crest cells [21]. The Human Protein Atlas reveals that the PGAP2 protein is ubiquitously expressed in almost all tissues, with a particularly notable presence in brain compartments (proteinatlas.org) [22].

Hypomorphic *Pgap2* mutant mice, generated via ENU mutagenesis, exhibit the Clpex phenotype (cleft lip, cleft palate, edema, and exencephaly) due to apoptosis of neural crest cells (NCCs) and the cranial neuroepithelium. Impaired FOLR1 receptor trafficking identified in Clpex mutants would inhibit folate uptake, suggesting potential rescue of the Clpex phenotype through folinic acid supplementation. Indeed, dietary folinic acid supplementation rescued the early embryonic lethal phenotype of *Folr1*-/- mice, enabling their survival to adulthood and partially rescuing the cleft lip phenotype in Clpex mutants [21].

Compound heterozygous *PGAP2* mutations were identified in a female patient presenting with developmental delay (DD), ID, speech delay, epilepsy, and hyperphosphatasia. Cerebrospinal fluid (CSF) analysis revealed low levels of pyridoxal phosphate (PLP) and 5-methyltetrahydrofolate (5-MTHF). Based on the reported effective management of untreatable seizures in hyperphosphatasia with mental retardation syndrome (HPMRS) patients with low serum PLP through pyridoxine supplementation, a combined supplementation of pyridoxine and folinic acid was administered. This treatment successfully normalized both metabolites in the female patient, resulting in developmental progress and improved speech. These findings suggest that low CSF levels of PLP and 5-MTHF may serve as characteristic features of HPMRS3, which show potential responsiveness to treatment with pyridoxine and folinic acid [19].

To date, only 14 biallelic *PGAP2* mutations in 20 patients have been documented revealing variable degrees of DD and ID [3, 5, 7, 9–11, 19, 23–26] (Figure 1 and Supplementary Table 1). However, when compared to other HPMRS types, these patients exhibit a lower incidence of epilepsy, hypotonia, dysmorphisms, and organ anomalies [19]. According to published patient reports, it has been observed that 95% of patients with *PGAP2* mutations have ID, with three individuals reported as having mild ID (*n* = 19/20). Additionally, 95% of patients have DD (*n* = 20/21), with three individuals reported as having mild DD. Seizures/epilepsy were found in 57% of patients (*n* = 12/21), while hearing impairment was present in 12.5% of patients (*n* = 2/16) [5, 19]. In the present study, two male siblings with a homozygous Arg238Pro variant in family 1 exhibited hyperphosphatasia, bilateral hearing loss, language/speech delay, facial dysmorphism, macrocephaly, severe ID, tapering fingers, camptodactyly, brachydactyly, and syndactyly. In contrast, two female siblings with the compound heterozygous N177S and G216R variants in family 2 had refractory epilepsy, craniofacial anomalies, severe ID, tapering fingers, brachydactyly, clino/camptodactyly, and syndactyly, but no hearing impairment. Hearing impairment has been reported in two independent consanguineous families with *PGAP2* mutations (Supplementary Table 1). In the first family, a Turkish boy exhibited sensorineural hearing loss, seizures, ID, hyperphosphatasia, microcephaly, facial dysmorphism, heart defect, tapering fingers, and cleft palate. He carried a homozygous mutation (c.563T>C, p.Leu188Ser) in *PGAP2* (Figure 1 and Supplementary Table 1) [10]. In the second family, a Saudi Arabian boy and his elder sister experienced poor hearing, epilepsy, ID, hyperphosphatasia, and microcephaly. They carried a homozygous mutation (c.191C>T, p.Ala64Val) in *PGAP2* (Figure 1 and Supplementary Table 1) [11].

As previously mentioned, microcephaly has been reported as a partial clinical feature in three patients with *PGAP2* mutations from two distinct families: a boy in a consanguineous Turkish family and two siblings (one boy and one girl) in a consanguineous Saudi family (Supplementary Table 1). Notably, macrocephaly, which has not been reported previously, was observed in two affected males in our Pakistani family 1 with *PGAP2* mutation (Figure 3(a)). Interestingly, macrocephaly has been reported in patients with mutations in *PIGA* [27], *PIGC* [28], and *PIGM* [29], which are involved in the initial stages of GPI anchor biosynthesis. Furthermore, this cranial phenotype has also been documented in an individual with *PIGU* mutation [30]. PIGU functions as a subunit of the GPI transamidase complex, responsible for attaching GPI anchors to proteins during their maturation.

This suggests that disruption in the early steps of GPI anchor biosynthesis can contribute to the development of macrocephaly. In contrast, PGAP2 is involved in the posttranslational modification of GPI-anchored proteins (GPI-APs), highlighting its distinct role in the later stages of the GPI anchor pathway.

Our four patients from two unrelated families exhibit common digital anomalies, including tapering fingers, brachydactyly, camptodactyly, and syndactyly (Figure 3). While tapering fingers were previously reported in the consanguineous Turkish boy with a *PGAP2* mutation [10], the remaining digital phenotypes (brachydactyly, camptodactyly, and syndactyly) have not been reported in patients with *PGAP2* mutations (Supplementary Table 1). Therefore, our patients, showcasing the novel cranial phenotype of macrocephaly and these previously unreported digital anomalies, broaden the phenotypic spectrum of *PGAP2* mutations in HPMRS3.

The more severe phenotype observed in the two male siblings in family 1, in comparison to family 2, could be attributed to the potentially greater impact of the R238P substitution on protein function, as suggested by protein modeling and the high CADD score. A multiple protein sequence alignment was conducted, comparing PGAP2 with its ten vertebrate orthologs. Remarkably, the three amino acids affected by missense variants, namely, N177, G216, and R238, exhibit complete conservation in 10 vertebrate species, underscoring their crucial role in the gene's functionality and their participation in shared biological processes across these species (Figure 2(c)). The detrimental effects of the three amino acid substitutions were further supported by protein modeling (Figure 4).

It is noteworthy that *PGAP2*-associated HPMRS3 demonstrates a broader range of clinical features. Among the variants presented in this study, the homozygous variant c.713G>C (NM_014489.4), p.Arg238Pro (NP_055304.1) in isoform 1, has been previously reported in two families. In one of these families, which shares the same variant c.530G>C (NM_001256240.1), p.Arg177Pro (NP_001243169.1) in isoform 8, and the same Pakistani origin as our patients, four affected individuals exhibited severe ID (with an average IQ of 22) and no epilepsy, consistent with our findings [9].

However, DD, macrocephaly, digital anomalies, or hearing impairments found in our family 1 were not observed in that family [31], indicating certain inconsistencies [9]. In a separate report, the same variant, identified using the same RefSeq, was associated with a nonsyndromic ID in a multiplex family from Pakistan (JB Vincent, personal communication), suggesting ID as the sole clinical feature [24] (Supplementary Table 1). These findings emphasize that even with the presence of the same variants within the same ethnicity, discordant phenotypes can emerge, which may be explained by indicating the potential influence of other modifier genes in shaping the observed outcomes. Intriguingly, the partial phenotype observed in our patients—including intellectual disability, hearing loss, facial dysmorphism, and camptodactyly—significantly overlaps with those seen in a patient with Takenouchi-Kosaki syndrome and a heterozygous c.191A>G (p.Tyr64Cys) variant in *CDC42* [32].

To our knowledge, neither c.530A>G, p.Asn177Ser, nor c.646G>A, p.Gly216Arg, observed in family 2 has been previously reported in the literature, expanding the genotypic spectrum of *PGAP2*. Accordingly, the compound heterozygous presence of these variants leads to epilepsy, ID, craniofacial anomalies, and hyperphosphatasia. Among these phenotypes, epilepsy has been relatively less frequently observed in the literature [5, 19]. This represents the fifth case of a nonconsanguineous family with compound heterozygous variants in *PGAP2*, adding to the previously reported four families (Supplementary Table 1).

Despite known limitations and potential pitfalls, ES remains a valuable tool for identifying causative variants in Mendelian diseases. This is especially crucial in clinically heterogeneous diseases where achieving a molecular diagnosis is of utmost importance. Therefore, ES should be strongly recommended as the first-tier clinical diagnostic test for NDDs [33].

## 5. Conclusion

This study provides clinical and genetic insights into HPMRS3, highlighting the diverse clinical manifestations associated with variants in *PGAP2*. It underscores the significance of the GPI anchor biosynthesis pathway and its role in NDDs. Further research is needed to elucidate the functional consequences of these variants and their impact on GPI-APs and neurological development.

## Figures and Tables

**Table 1:** Nomenclatures and minor allele frequencies of *PGAP2* variants in isoform 1 in two families.

Family	Family 1	Family 2	Family 2
Origin	Pakistani	German	German
PGAP2 variant (NM_014489.4)	c.713G>C	c.530A>G	c.646G>A
Location (hg38)	11:3825024	11:3824064	11:3824314
Location (hg19)	11:3846254	11:3845294	11:3845544
Variant type	Missense	Missense	Missense
Inheritance	Homozygous	Compound heterozygous	Compound heterozygous
Protein effect (NP_055304.1)	p.Arg238Pro	p.Asn177Ser	p.Gly216Arg
Exon	6	4	5
Codons	cGc/cCc	aAc/aGc	Ggg/Agg
Existing variation	rs774843232	rs377757894	rs1064797152
gnomAD exomes_AF	3.58*e* − 05	0	3.98*E* − 03
gnomAD genomes_AF	6.57*E* − 03	0	6.57*E* − 03
gnomAD homozygotes	0	0	0
CADD_PHRED	23.6	19.64	22.0
VARSOME	Pathogenic	Uncertain significance	Uncertain significance
ACMG classification and interpretation	PS4, PM2, PP1, PP3, and PP4 pathogenic	PS4, PM2, PP1, PP3, and PP4 pathogenic	PS4, PM2, PP1, PP3, and PP4 pathogenic

AF: allele frequency.

## Data Availability

The variant data used to support the findings of this study have been deposited in the ClinVar repository and are under processing.

## Supplementary Materials

Supplementary Materials Supplementary Table 1 outlines one heterozygous, eight homozygous, and four compound heterozygous variants in *PGAP2*, each associated with distinct phenotypes across 12 ethnic groups. The nomenclature of these variants, as presented in 12 publications and this manuscript, has been described, considering that the referenced papers used two different isoforms of *PGAP2*.
