# Correction to “Macrocephaly and Digital Anomalies Expand the Phenotypic Spectrum of *PGAP2* Variants in Hyperphosphatasia with Impaired Intellectual Development Syndrome 3 (HPMRS3)”

**DOI:** 10.1155/humu/9836575

**Published:** 2025-10-28

S. Susgun, A. Ben-Mahmoud, F. Rüschendorf, et al., “Macrocephaly and Digital Anomalies Expand the Phenotypic Spectrum of *PGAP2* Variants in Hyperphosphatasia with Impaired Intellectual Development Syndrome 3 (HPMRS3),” *Human Mutation* 2024 (2024): 5518289, https://doi.org/10.1155/2024/5518289.

In the article titled “Macrocephaly and Digital Anomalies Expand the Phenotypic Spectrum of *PGAP2* Variants in Hyperphosphatasia with Impaired Intellectual Development Syndrome 3 (HPMRS3),” author “Solveig Schulz” was affiliated to “Zentrum für Humangenetik, Tübingen, Germany,” which is incorrect. The correct affiliation for this author appears below:

“Synlab Praxis Humangenetik, Jena, Germany”

We apologize for this error.

