# Supplementary material for: Macrocephaly and Digital Anomalies Expand the Phenotypic Spectrum of PGAP2 Variants in Hyperphosphatasia with Impaired Intellectual Development Syndrome 3 (HPMRS3)
Source: Hum Mutat. 2024 Jan 5;2024:5518289. doi: 10.1155/2024/5518289 (PMC11919034; doi:10.1155/2024/5518289)
Supplement: Supplementary Materials — Supplementary Table 1 outlines one heterozygous, eight homozygous, and four compound heterozygous variants in PGAP2, each associated with distinct phenotypes across 12 ethnic groups. The nomenclature of these variants, as presented in 12 publications and this manuscript, has been described, considering that the referenced papers used two different isoforms of PGAP2. [file 5518289.f1.docx]

**Supplementary Table 1.** A total of 16 reported and novel variants identified in two isoforms of *PGAP2*

| **PGAP2 Variants** | | **Zygosity** | **Ethnicity** | **Detailed Phenotype** | **Reference** |
| --- | --- | --- | --- | --- | --- |
| **Isoform 8**  [**NM_001256240.2**](https://www.ncbi.nlm.nih.gov/nucleotide/NM_001256240.2)  [**NP_001243169.1**](https://www.ncbi.nlm.nih.gov/protein/NP_001243169.1) | **Isoform 1**  **NM_014489.4**  [**NP_055304.1**](https://mutalyzer.nl/normalizer/NM_014489.4(NP_055304.1):p.(Arg238Pro)) |  |  |  |  |
| c.2T>G, p.M1?  c.221G>A, p.R74H | c.2T>G, p.M1?  c.404G>A, [p.R135H](https://mutalyzer.nl/normalizer/NM_014489.4(NP_055304.1):p.(Arg135His)) | compound heterozygous | Polish | flat occiput and pectus excavatum, epilepsy, no speech, hyporeflexia, hypotonia, hyperthermia, progressive  neurological deterioration, hyperphosphatasemia. | (Jezela-Stanek et al., 2016) |
|  |  |  |  | psychomotor retardation, generalized weakness, seizures, sustained hyperphosphatasaemia | (Pronicka et al., 2016) |
| c.46C>T, p.R16W  c.479C>T, p.T160I | c.46C>T, p.R16W  c.662C>T, [p.T221I](https://mutalyzer.nl/normalizer/NM_014489.4(NP_055304.1):p.(Thr221Ile)) | compound heterozygous | Finnish | febrile seizure, epilepsy, subtle facial dysmorphism, mild ID, tapering fingers, hyperphosphatasia | (Krawitz et al., 2013) |
| c.380T>C, p.L127S | c.563T>C, [p.L188S](https://mutalyzer.nl/normalizer/NM_014489.4(NP_055304.1):p.(Leu188Ser)) | homozygous | Turkish | median cleft palate, atrial septal  defect, hypoplasia of the corpus callosum, marked ID, microcephaly, facial dysmorphism, [brachytelephalangy, tapering fingers, hearing impairment, hyperphosphatasia](https://en.wiktionary.org/wiki/brachytelephalangy) |  |
| c.103del, p.L35Sfs*90,  c.134A>G, p.H45R | c.103del, p.L35Sfs*90,  c.134A>G, p.H45R | compound heterozygous | Afro-Caribbean | global developmental delay, speech delay, precocious puberty, esotropia and myopic astigmatism, and hyperphosphatasia | (Messina et al., 2023) |
| c.165+5954C>T | c.191C>T, p.A64V | homozygous | Saudi Arabian | developmental delay, ID, epilepsy, poor hearing, microcephaly, hyperphosphatasia | (Naseer et al., 2016) |
| c.220C>T, p.R74C | c.403C>T, [p.R135C](https://mutalyzer.nl/normalizer/NM_014489.4(NP_055304.1):p.(Arg135Cys)) | homozygous | Jordanian | nystagmus, global developmental delay, aganglionic megacolon, hyperphosphatasia | (Froukh et al., 2020) |
| c.284A>G, p.Y95C | c.467A>G, p.Y156C | homozygous | Guatemalan (personal communication with Miles Thompson) | hyperphosphatasia and neurologic  deficit with additional homozygous *PGAP3* VUS | (Thompson et al., 2023) |
| c.296A>G, p.Y99C | c.479A>G, [p.Y160C](https://mutalyzer.nl/normalizer/NM_014489.4(NP_055304.1):p.(Tyr160Cys)) | homozygous | Syrian | fetal hypokinesia, development delay, severe ID, hyperphosphatasia, hypotonia, strabismus, sleep disturbance, absence  seizures, cerebral atrophy and increased gyration, Dandy‐Walker malformation, short stature | (Hansen et al., 2013; Reuter et al., 2017) |
| c.347A>G, p.N116S  c.463G>A, p.G155R | c.530A>G, p.N177S  c.646G>A, p.G216R | compound heterozygous | German | prominent forehead, facial dysmorphism, epilepsy, tapering fingers, brachydactyly, camptodactyly, and syndactyly, hyperphosphatasia | This study |
| c.530G>C, p.R177P | c.713G>C, [p.R238P](https://mutalyzer.nl/normalizer/NM_014489.4(NP_055304.1):p.(Arg238Pro)) | homozygous | Pakistani | Severe ID, macrocephaly, hearing loss, facial dysmorphism, tapering fingers, brachydactyly, camptodactyly, and syndactyly, hyperphosphatasia | This study |
|  |  |  | Pakistani | severe ID,  hyperphosphatasia, absence  seizures | (Hansen et al., 2013) |
|  |  |  | Pakistani  (personal communication with JB Vincent) | Non-syndromic ID | (Harripaul et al., 2018) |
| c.554G>A, p.R185Q | c.737G>A, [p.R246](https://mutalyzer.nl/normalizer/NM_014489.4(NP_055304.1):p.(Arg246Gln))Q | heterozygous  (carriers) | Bedouin | learning disability without ID and mild hyperphosphatasia | (Perez et al., 2017) |
|  |  | homozygous |  | ID, speech delay, behavioral problems, seizures, depression, enuresis, hyperphosphatasia. |  |
| c.698C>T, p.T233M | c.881C>T, [p.T294M](https://mutalyzer.nl/normalizer/NM_014489.4(NP_055304.1):p.(Thr294Met)) | homozygous | American | Mabry syndrome, hyperphosphatasia with  neurologic deficit ((HPMRS), developmental disability, seizures, brachytelephalangy | (Thompson et al., 2020) |

ID: intellectual disability

**References**

Froukh, T., Nafie, O., Al Hait, S. A. S., Laugwitz, L., Sommerfeld, J., Sturm, M., Baraghiti, A., Issa, T., Al-Nazer, A., Koch, P. A., Hanselmann, J., Kootz, B., Bauer, P., Al-Ameri, W., Abou Jamra, R., Alfrook, A. J., Hamadallah, M., Sofan, L., Riess, A., . . . Buchert, R. (2020). Genetic basis of neurodevelopmental disorders in 103 Jordanian families. *Clin Genet*, *97*(4), 621-627. <https://doi.org/10.1111/cge.13720>

Hansen, L., Tawamie, H., Murakami, Y., Mang, Y., ur Rehman, S., Buchert, R., Schaffer, S., Muhammad, S., Bak, M., Nothen, M. M., Bennett, E. P., Maeda, Y., Aigner, M., Reis, A., Kinoshita, T., Tommerup, N., Baig, S. M., & Abou Jamra, R. (2013). Hypomorphic mutations in PGAP2, encoding a GPI-anchor-remodeling protein, cause autosomal-recessive intellectual disability. *Am J Hum Genet*, *92*(4), 575-583. <https://doi.org/10.1016/j.ajhg.2013.03.008>

Harripaul, R., Vasli, N., Mikhailov, A., Rafiq, M. A., Mittal, K., Windpassinger, C., Sheikh, T. I., Noor, A., Mahmood, H., Downey, S., Johnson, M., Vleuten, K., Bell, L., Ilyas, M., Khan, F. S., Khan, V., Moradi, M., Ayaz, M., Naeem, F., . . . Vincent, J. B. (2018). Mapping autosomal recessive intellectual disability: combined microarray and exome sequencing identifies 26 novel candidate genes in 192 consanguineous families. *Mol Psychiatry*, *23*(4), 973-984. <https://doi.org/10.1038/mp.2017.60>

Jezela-Stanek, A., Ciara, E., Piekutowska-Abramczuk, D., Trubicka, J., Jurkiewicz, E., Rokicki, D., Mierzewska, H., Spychalska, J., Uhrynowska, M., Szwarc-Bronikowska, M., Buda, P., Said, A. R., Jamroz, E., Rydzanicz, M., Ploski, R., Krajewska-Walasek, M., & Pronicka, E. (2016). Congenital disorder of glycosylphosphatidylinositol (GPI)-anchor biosynthesis--The phenotype of two patients with novel mutations in the PIGN and PGAP2 genes. *Eur J Paediatr Neurol*, *20*(3), 462-473. <https://doi.org/10.1016/j.ejpn.2016.01.007>

Krawitz, P. M., Murakami, Y., Riess, A., Hietala, M., Kruger, U., Zhu, N., Kinoshita, T., Mundlos, S., Hecht, J., Robinson, P. N., & Horn, D. (2013). PGAP2 mutations, affecting the GPI-anchor-synthesis pathway, cause hyperphosphatasia with mental retardation syndrome. *Am J Hum Genet*, *92*(4), 584-589. <https://doi.org/10.1016/j.ajhg.2013.03.011>

Messina, M., Manea, E., Cullup, T., Tuschl, K., & Batzios, S. (2023). Hyperphosphatasia with mental retardation syndrome 3: Cerebrospinal fluid abnormalities and correction with pyridoxine and Folinic acid. *JIMD Rep*, *64*(1), 42-52. <https://doi.org/10.1002/jmd2.12347>

Naseer, M. I., Rasool, M., Jan, M. M., Chaudhary, A. G., Pushparaj, P. N., Abuzenadah, A. M., & Al-Qahtani, M. H. (2016). A novel mutation in PGAP2 gene causes developmental delay, intellectual disability, epilepsy and microcephaly in consanguineous Saudi family. *J Neurol Sci*, *371*, 121-125. <https://doi.org/10.1016/j.jns.2016.10.027>

Perez, Y., Wormser, O., Sadaka, Y., Birk, R., Narkis, G., & Birk, O. S. (2017). A Rare Variant in PGAP2 Causes Autosomal Recessive Hyperphosphatasia with Mental Retardation Syndrome, with a Mild Phenotype in Heterozygous Carriers. *Biomed Res Int*, *2017*, 3470234. <https://doi.org/10.1155/2017/3470234>

Pronicka, E., Piekutowska-Abramczuk, D., Ciara, E., Trubicka, J., Rokicki, D., Karkucinska-Wieckowska, A., Pajdowska, M., Jurkiewicz, E., Halat, P., Kosinska, J., Pollak, A., Rydzanicz, M., Stawinski, P., Pronicki, M., Krajewska-Walasek, M., & Ploski, R. (2016). New perspective in diagnostics of mitochondrial disorders: two years' experience with whole-exome sequencing at a national paediatric centre. *J Transl Med*, *14*(1), 174. <https://doi.org/10.1186/s12967-016-0930-9>

Reuter, M. S., Tawamie, H., Buchert, R., Hosny Gebril, O., Froukh, T., Thiel, C., Uebe, S., Ekici, A. B., Krumbiegel, M., Zweier, C., Hoyer, J., Eberlein, K., Bauer, J., Scheller, U., Strom, T. M., Hoffjan, S., Abdelraouf, E. R., Meguid, N. A., Abboud, A., . . . Abou Jamra, R. (2017). Diagnostic Yield and Novel Candidate Genes by Exome Sequencing in 152 Consanguineous Families With Neurodevelopmental Disorders. *JAMA Psychiatry*, *74*(3), 293-299. <https://doi.org/10.1001/jamapsychiatry.2016.3798>

Thompson, M. D., Knaus, A. A., Barshop, B. A., Caliebe, A., Muhle, H., Nguyen, T. T. M., Baratang, N. V., Kinoshita, T., Percy, M. E., Campeau, P. M., Murakami, Y., Cole, D. E., Krawitz, P. M., & Mabry, C. C. (2020). A post glycosylphosphatidylinositol (GPI) attachment to proteins, type 2 (PGAP2) variant identified in Mabry syndrome index cases: Molecular genetics of the prototypical inherited GPI disorder. *Eur J Med Genet*, *63*(4), 103822. <https://doi.org/10.1016/j.ejmg.2019.103822>

Thompson, M. D., Li, X., Spencer-Manzon, M., Andrade, D. M., Murakami, Y., Kinoshita, T., & Carpenter, T. O. (2023). Excluding Digenic Inheritance of PGAP2 and PGAP3 Variants in Mabry Syndrome (OMIM 239300) Patient: Phenotypic Spectrum Associated with PGAP2 Gene Variants in Hyperphosphatasia with Mental Retardation Syndrome-3 (HPMRS3). *Genes (Basel)*, *14*(2). <https://doi.org/10.3390/genes14020359>
